# Determination of neutrophil-to-lymphocyte ratio, platelet-to-lymphocyte ratio and systemic immune-inflammation index in dogs with leptospirosis

**DOI:** 10.1007/s11259-024-10469-y

**Published:** 2024-09-10

**Authors:** A. Durán-Galea, J. I. Cristóbal-Verdejo, B. Macías-García, P. Nicolás-Barceló, R. Barrera-Chacón, P. Ruiz-Tapia, M. C. Zaragoza-Bayle, F. J. Duque-Carrasco

**Affiliations:** 1 Universidad de Extremadura, Hospital Clínico Veterinario, Avenue University n/n, 10003 Cáceres, Spain; 2 Department of Animal Medicine, University of Extremadura, Avenue University n/n, 10003 Cáceres, Spain

**Keywords:** Canine leptospirosis, Inflammation ratios, Neutrophil-to-lymphocyte ratio, Platelet-to-lymphocyte ratio, Systemic immune-inflammation index, Pulmonary hemorrhagic syndrome

## Abstract

Leptospirosis is a globally distributed zoonosis with multisystemic involvement in canine species, capable of causing a pulmonary hemorrhagic syndrome (LPHS) in the most severe cases. In humans, the neutrophil to lymphocyte ratio (NLR), platelets to lymphocytes (PLR) and systemic immune-inflammation index (SII) have been described as predictors of morbidity and mortality in various pathologies, but no such studies have been developed for canine leptospirosis. Hence, we aimed to assess the usefulness of NLR, PLR and SII in dogs affected with leptospirosis, focusing on those that died or survived after hospitalization, whether or not they developed LPHS. The leptospirosis group was composed by 36 dogs while the control group consisted of 32 healthy dogs. The NLR, associated with inflammation, demonstrated a threefold or greater increase in all leptospirosis groups compared to the control group (median 2.44 ± 1.66) (developing or not LPHS). Dogs that died (median 67.78 ± 158.67), developed LHPS (median 85.17 ± 143.77), or both developed LHPS and died (median 67.78 ± 155,14) had a lower PLR in comparison to the control group (median 101,82 ± 53,75) and the rest of groups, but no statistically significant differences were observed (*p* > 0.05). The SII was higher in leptospirosis-affected dogs that survived (median 1356,92 ± 2726,29) and statistically significant differences were observed in those who did not develop LPHS (median 1770,41 ± 2630,77; *p* < 0.05) compared to the control group (median 555,21 ± 313,26). Our data shows that NLR may be used as inflammation indicator, while more studies are needed for PLR and SII in canine leptospirosis.

**Supplementary Information:**

The online version contains supplementary material available at 10.1007/s11259-024-10469-y.

## Introduction

Leptospirosis is a global zoonosis caused by spirochetal bacteria belonging to the genus *Leptospira*, which affects a wide range of mammalian species. Currently, it is considered an emerging disease in both humans and dogs and a serious public health issue. Most cases of leptospirosis in dogs result from infections by P1-virulent species such as *Leptospira interrogans*,* Leptospira borgpetersenii*, and *Leptospira kirschneri* (Reagan and Sykes 2019; Schuller et al. 2015; Sykes et al. 2011, 2023).

Leptospirosis is a multi-systemic disease that primarily affects the kidneys and liver, but it can also involve other organs. Among the affected organs are the lungs, spleen, endothelial cells, uvea/retina, skeletal and heart muscles, meninges, pancreas, and the genital tract a (Reagan and Sykes 2019; Schuller et al. 2015; Zaragoza et al. 2003).

One particularly dangerous complication of canine leptospirosis is leptospiral pulmonary hemorrhage syndrome (LPHS), which poses a life-threatening risk (Kohn et al. 2010; Schuller et al. 2015). This syndrome is characterized by severe respiratory distress and quick intra alveolar hemorrhage in the absence of inflammatory cell infiltration (Sonderegger et al. 2021). The pathogenesis of LPHS is poorly understood but the causes leading to pulmonary bleeding are likely multifactorial, including immunologic mechanisms, vascular damage caused by the leptospiral toxins or the induction of disseminated intravascular coagulopathy (Klopfleisch et al. 2010; Kohn et al. 2010; Sykes et al. 2023). Similar to previous reports in humans, leptospirosis-associated pulmonary syndrome has recently been documented in European dogs. This condition presents high morbidity and lethality rates, reaching approximately 70–77% and 44–48%, respectively (Maissen-Villiger et al. 2016).

The diagnosis of leptospirosis typically requires a combination of clinical signs, a history of exposure to potentially contaminated environments, and specific laboratory tests such as serology or polymerase chain reaction (PCR) assays or the microscopic agglutination test (MAT). Early detection and prompt treatment are crucial to improve the prognosis of the patients (Reagan and Sykes 2019; Schuller et al. 2015; Sykes et al. 2023). Although abnormalities in blood cell count, serum biochemistry, and urinalysis have been observed, none of these changes can provide a definitive diagnosis of *Leptospira spp*. per se.

Interestingly, the neutrophil-to-lymphocyte ratio (NLR), platelet-to-lymphocyte ratio (PLR) and systemic immune-inflammation index (SII) can be used as predictors of morbidity and mortality in critically ill human patients with oncological and/or cardiovascular diseases (Benvenuti et al. 2020; Kang et al. 2014; Salciccioli et al. 2015; Wang et al. 2022; Yang et al. 2020). These ratios are indicative of systemic inflammation and can offer valuable insights into the patient’s overall health status (Ji et al. 2021). Some studies have suggested that SII serves as a marker of chronic inflammation, indicated by increased neutrophil and platelet counts and decreased lymphocyte counts (Ye et al. 2023). In cases of canine leptospirosis, patients typically present with neutrophilia in 27–94% of cases, lymphopenia in 2–29% of cases, and thrombocytopenia in 14–73% of cases, representing the most prevalent hematological changes. Consequently, the SII in these patients may be significantly affected (Sykes et al. 2023).

In dogs, these ratios have been evaluated in various conditions such as congestive heart failure (CHF) secondary to myxomatous mitral valve degeneration, septic peritonitis, pancreatitis, chronic inflammatory enteropathy, or different types of tumors among others. Furthermore, it has been demonstrated that higher values of NLR are associated with a more severe inflammatory status, a higher degree of malignancy in case of neoplasia and, in general, with an overall poorer prognosis (Cristóbal et al. 2022; DeProspero et al. 2023; Hodgson et al. 2018; Neumann 2021; Park et al. 2022; Pierini et al. 2019).

Therefore, the NLR, PLR, and SII can be useful and cost-effective tools for veterinarians to assess the severity of inflammation and predict the outcome in critically ill dogs with different diseases. Besides, early recognition of systemic inflammation and its severity can aid in the timely implementation of appropriate treatments, potentially improving patient outcomes (Cristóbal et al. 2022; DeProspero et al. 2023; Hodgson et al. 2018; Macfarlane et al. 2016a, b; Neumann 2021; Park et al. 2022; Pierini et al. 2019).

Hence, in view of the previous literature, the objectives of the present study were to assess variations in the NLR, PLR and SII in different groups of dogs affected with *Leptospira spp*. when compared against a healthy group of dogs.

## Materials and methods

### Animals

The study included a first retrospective part followed by a prospective one; clinical cases between 1st of January of 2012 and 31st of March of 2023 have been carefully reviewed and included. All owners approved and signed an informed consent form.

All animals were divided into different groups according to their health status.

A group of healthy animals or control group (CG), composed by 32 dogs presented for annual check-ups or elective sterilization at the Internal Medicine Department of the Veterinary Teaching Hospital (School of Veterinary Medicine of the University of Extremadura, Spain) were included. In all dogs, normal physical examination, complete blood count and serum biochemical analysis were performed, and no alterations were obtained. All dogs were tested negative for Anaplasmosis, Ehrlichiosis, Leishmaniasis and, Heartworm disease (Uranotest Quattro, Uranovet, Spain).

A group with leptospirosis (36 dogs not vaccinated for *Leptospira spp.*) was diagnosed by antibody tests based on microscopic agglutination (MAT) and/or positive blood/urine Leptospira-specific PCR according to the updated ACVIM consensus (Sykes et al. 2023). 2 ml of drain blood and 2 ml of urine were collected for *Leptospira spp.* testing. Sick dogs were tested negative for *Anaplasma platys*, *Ehrlichia canis*, *Leishmania infantum* and, *Dirofilaria immitis* (Uranotest Quattro, Uranovet, Spain). These dogs were subsequently divided based on their survival after the initial hospitalization (died or lived, DL or LL, respectively). Furthermore, the dogs were classified based on whether they developmed LPHS (LPHS or NO LPHS, respectively). Among the LPHS-positive patients, they were also classified depending on if they died (DPHS) or lived (LPHS).

### Laboratory analysis

EDTA-K3 was used as anticoagulant for hematology (1 ml of sample). Blood samples were analyzed in an automatic analyzer (BC-5300 Vet, Mindray, Guangdong (China)) and a complete study of the erythrocyte, leukocyte and platelet series was conducted. Blood smears (Diff quick staining, Química Clínica Aplicada S.A., Tarragona (Spain)) and manual counting at 40x of the entire slide were performed in all dogs.

For blood biochemistry analysis, sodium heparin was used as anticoagulant to prevent blood clotting (2 ml of blood is extracted). Plasma was obtained after centrifugation at 300 g for 10 min (between 0.5 and 1 ml) and was used to determine the following parameters: urea, creatinine, phosphorus, total proteins and albumin; all samples were analyzed in an automatic analyzer (Saturno 100, VetCrony Instruments, Roma (Italy)), using specific commercial kits for each parameter (Spinreact Laboratories, Barcelona (Spain)). Globulin concentration was calculated by subtracting albumin concentration from that of total protein. The hematology and biochemistry analysis were performed immediately after sample obtention.

### Ratio calculation

Using hematological values of neutrophils, lymphocytes and platelets a series of ratios were calculated: NLR, PLR and SII. The NLR was calculated as total neutrophil to total lymphocyte count ratio as previously described by Arbel et al. (2012) and the PLR was calculated as the ratio of the platelet count to the lymphocyte count as previously described Kurtul et al. (2014). The SII was developed based on the platelet counts and NLR (SII, platelets count * neutrophil/lymphocyte ratio) as described Yang et al. (2020).

### Statistical modelling

The statistical study was carried out using SigmaPlot 14.5 Software and the text has been written with Microsoft Word 2016 Software. A Shapiro-Wilk normality test was performed revealing a non-gaussian distribution of the data. Hence, a Kruskal-Wallis test followed by a Dunn’s post-hoc test were conducted for all pairwise comparisons. Statistical significance was assumed when *p* < 0,05. Data are presented as median ± interquartile range.

## Results

The leptospirosis group was composed by 36 dogs of different breeds (mainly crossbreeds), including 18 males (median and interquartile range of 6,2 ± 2,3 years) and 18 females (median and interquartile range of 7 ± 2,1 years).

The group of sick dogs was classified based on different criteria, including survival (21 patients lived and 15 died); the onset of pulmonary hemorrhagic syndrome (21 dogs) or not (15 animals); and the survival of those who developed pulmonary hemorrhagic syndrome (10 dogs) or not (11 dogs). If the patient died, the death occurred between 2 h and 72 h after admission at the hospital.

**Fig. 1:**
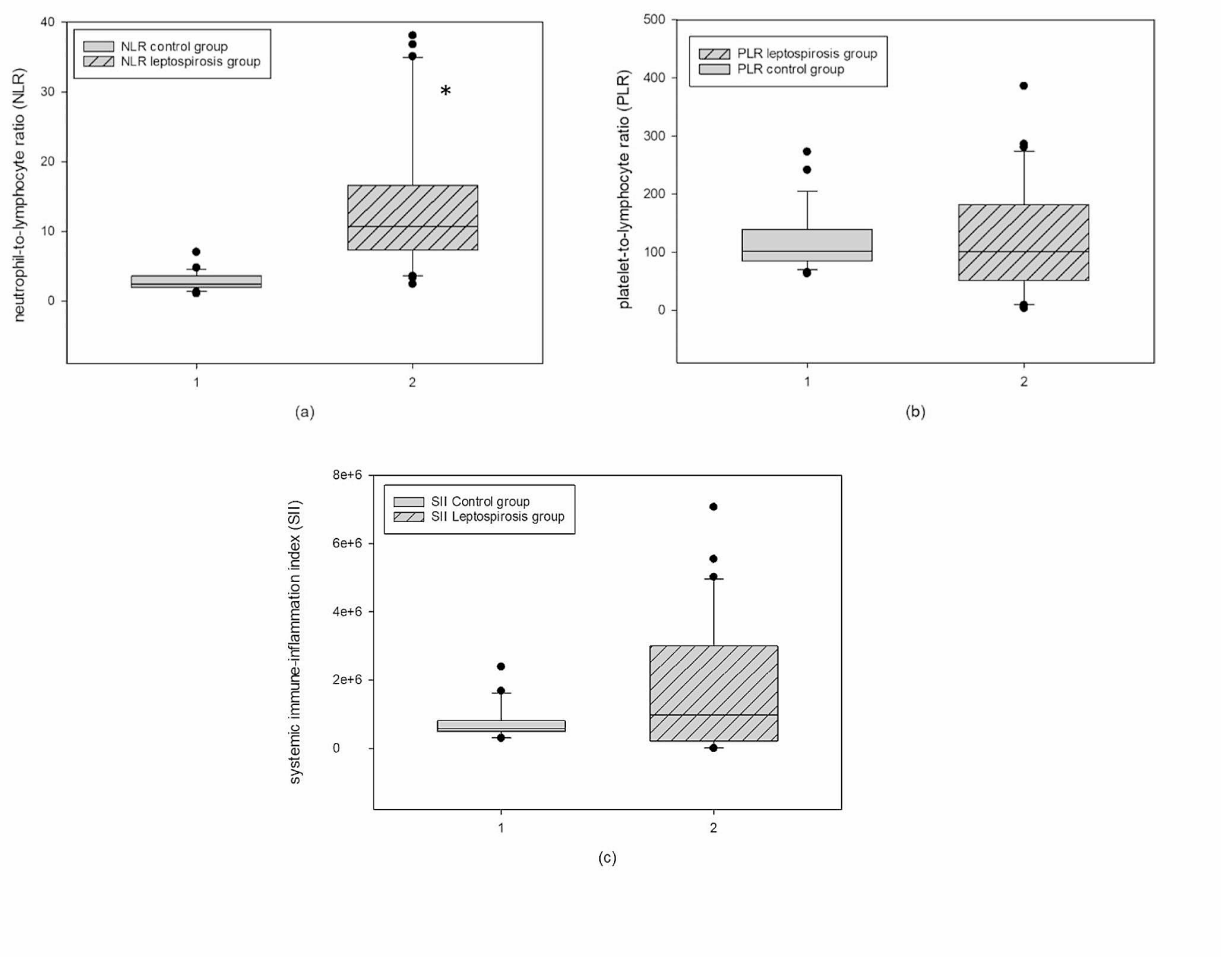
NLR, PLR and SII in dogs affected or not with leptospirosis. The bars show the values as median ± interquartile range of neutrophil-to-lymphocyte ratio (NLR) (**a**), platelet-to-lymphocyte ratio (PLR) (**b**) and systemic immune-inflammation index (SII) (**c**) in control group and leptospirosis group

In Fig. 1, the values of NLR, PLR, and SII for both healthy dogs and dogs with leptospirosis are presented. Statistical analysis showed that there are statistically significant differences (*p* < 0,05) in the NLR between the healthy dogs (2,44 ± 1,66) and the dogs affected with leptospirosis (10,70 ± 9,33; Fig. 1a). However, no statistically significant differences were observed in PLR and SII between the two groups (Fig. 1b and c). The SII value in leptospirosis dogs is higher than healthy dogs (978037,81 ± 2778946,83 and 555214,55 ± 313256,54, respectively). The PLR value was similar in healthy (101,82 ± 53,75) and leptospirosis dogs (101,25 ± 130,43).

The alterations in NLR, PLR and SII found in dogs dying of leptospirosis (DL) and dogs living after being hospitalized due to leptospirosis (LL) are detailed in Table 1. Statistically significant differences were observed in both sick groups compared to the control group, only in the NLR.

**Table 1:** NLR, PLR and SII in control group and leptospirosis affected dogs that lived or died after hospitalization

	NLR	PLR	SII (x10^3^)
Control group (*N* = 32)	2,44 ± 1,66	101,82 ± 53,75	555,21 ± 313,26
Died leptospirosis (*n* = 15)	10,99 ± 8,98*	67,78 ± 158,67	553,76 ± 1906,017
Lived leptospirosis (*n* = 21)	10,41 ± 10,89*	108,79 ± 108,71	1356,92 ± 2726,29

*NLR* neutrophil-to-lymphocyte ratio, *PLR* platelet-to-lymphocyte ratio, *SII* systemic immune-inflammation index

* Statistically significant differences (*p* < 0,05) respect control group

Interestingly, if dogs developed LPHS, the NLR sowed statistically significant differences compared to CG and NO LPHS groups (Table 2; *p* < 0,05). The SII showed statistically significant differences compared to the CG and between the dogs that developed LPHS and those that did not (Table 2).

**Table 2:** NLR, PLR and SII in leptospirosis infected dogs that developed or not hemorrhagic pulmonary syndrome

	NLR	PLR	SII (x10^3^)
Control (*n* = 32)	2,44 ± 1,66	101,82 ± 53,75	555,21 ± 313,26
LPHS (*n* = 21)	12,50 ± 9,13*	85,17 ± 143,77	614,45 ± 1828,06
NO LPHS (*n* = 15)	8,93 ± 10,44*	108,79 ± 114,10	1770,41 ± 2630,77*^1^

*NLR* neutrophil-to-lymphocyte ratio, *PLR* platelet-to-lymphocyte ratio, *SII* systemic immune-inflammation index, *LPHS* pulmonary hemorrhagic syndrome

* Statistically significant differences (*p* < 0,05) respect control group. Within columns, ^1^represents statistically significant differences compared to LPHS (*p < *0,05)

In Table 3, despite an observed increase in the NLR in both the LPHS and DPHS groups compared to the CG, there were no statistically significant differences among groups (*p* > 0,05).

**Table 3:** NLR, PLR and SII in control and LPHS groups that died or lived

	NLR	PLR	SII (x10^3^)
Control (*n* = 32)	2,44 ± 1,66	101,82 ± 53,75	555,21 ± 313,26
DPHS (*n* = 11)	10,99 ± 7,33	67,78 ± 155,14	553,76 ± 633,58
LPHS (*n* = 10)	13,53 ± 13,78	114,88 ± 164,43	709,77 ± 2822,53

*NLR* neutrophil-to-lymphocyte ratio, *PLR* platelet-to-lymphocyte ratio, *SII* systemic immune-inflammation index, *DPHS* hemorrhagic pulmonary syndrome died, *LPHS* hemorrhagic pulmonary syndrome lived

No statistically significant differences were observed in the PLR for any group (*p* > 0,05), but dogs that died or developed LPHS presented a lower PLR than the rest of groups. Among the animals that died, (7 out of 15 dogs) displayed moderate to severe thrombocytopenia, with an average platelet count of 107 ± 171 × 10^3^/µl. The average platelet count in surviving patients with LPHS was 159 ± 134 × 10^3^K/µl. No statistically significant differences were observed (*p* > 0,05).

## Discussion

This study aimed to determine the usefulness of NLR, PLR, and SII in leptospirosis – infected dogs. Although there are not gold standard values for these ratios, a previous report established range reference values for healthy dogs for NLR (0,74 − 5,62), PLR (56,41–198,02) and SII (52,93-1503) (Cristóbal et al. 2022). Hence, in the present work, the values of these indexes in the control group fell within the established ranges for healthy individuals.

Recent studies in human medicine have identified the NLR, PLR, and SII as important predictors of morbidity and mortality in critically ill patients (Benvenuti et al. 2020; Kang et al. 2014; Salciccioli et al. 2015; Wang et al. 2022; Yang et al. 2020; Zinellu et al. 2022). These parameters are part of a broader approach that uses hematological parameters to gauge systemic inflammation and immune response, which can significantly impact the patient’s prognosis.

In previous studies, elevated NLR has been associated with worse prognosis in various pathologies in dogs (Cristóbal et al. 2022; DeProspero et al. 2023; Hodgson et al. 2018; Macfarlane et al. 2016a, b; Neumann 2021; Park et al. 2022; Pierini et al. 2019), being suggestive of a heightened response to inflammatory insults. This raise in NLR suggests a relative immunosuppression secondary to continued innate immune system stimulation and downregulation of the adaptive immune system (Hodgson et al. 2018). In our study, an increase in NLR was observed in all leptospirosis groups compared to the control group (dying or living after hospitalization and those that developed LPHS or not). However, in dogs with LPHS, no significant differences were observed among patients despite their survival. This suggests that while NLR may be related to the severity of the disease (as indicated by the development of LPHS), it may not be directly predictive of the ultimate outcome (survival or death). It is important to note that in our study approximately half of the animals that developed LPHS survived (10/21), coinciding with the results obtained in humans in which 50% survival is reported (Haake and Levett 2015; Kohn et al. 2010; Maissen-Villiger et al. 2016; Marchiori et al. 2011). Hence, NLR can systematically and accurately reflect the degree of body inflammation and stress (Chen et al. 2023), typical of any infectious disease such as leptospirosis, but is not associated with patient´s mortality in dogs affected with *Leptospira spp.* unlike in humans in which NLR is a good predictor of mortality (Ji et al. 2021; Zinellu et al. 2022).Our findings could be explained by the different leukocyte patterns between dogs and humans and the presence of a stress leukogram, which is not reported in humans (Pierini et al. 2019).

In different studies performed in dogs with chronic inflammatory enteropathies and pancreatitis, the PLR decreased with clinical improvement (Cristóbal et al. 2022; Neumann 2021). Conversely, in our study, lower PLR values were observed in those groups where animals died of the disease or its complications (died leptospirosis, LPHS and DPHS), with values similar to healthy dogs in the remaining groups. This can be explained as in leptospirosis affected dogs, the initial activation of the inflammatory response causes an increase in platelet activation and function. Then, when the compensatory mechanisms are depleted, thrombocytopenia is triggered by platelet consumption due to activation, adhesion and aggregation to a stimulated vascular endothelium, cell phagocytosis, immune–mediated platelet lysis or splenic sequestration (Espadas-González et al. 2023; Schuller et al. 2015; Wang et al. 2022). This scenario is observed in our study as absolute platelet count results are lower in dogs that died compared to those that survived, as previously reported, and lymphocyte counts were also lower (Barthélemy et al. 2017). This may help to explain why the PRL ratio does not vary despite active leptospirosis infection. However, these observations contrast with previous studies in humans and dogs in which PLR can predict patient mortality (Cristóbal et al. 2022; Li et al. 2021; Neumann 2021; Zinellu et al. 2022);. These divergences may be attributed to the different pathologies under study, and thus, the PLR behavior could vary significantly. Hence our study suggests that PRL does not predict mortality of dogs affected with *Leptospira spp.* despite LPHS occurrence.

In the dogs studied, no increase was observed in the SII compared to the control group in the groups where the animals died. In these groups, the neutrophil count increases while those of lymphocytes and platelets decrease, which affected SII values. Thrombocytopenia, which is common in dogs infected with *Leptospira spp*., likely contributes to these lower SII values in final stages of the disease or more critical phases (died or LPHS development) (Schuller et al. 2015). These results do not agree with other authors where high values of SII were associated with a worse prognosis in human patients affected with cancer and cardiovascular diseases (Huang et al. 2019; Xia et al. 2023). In veterinary medicine, decreases in this index have been observed after improvement of the disease or damage in chronic inflammatory enteropathy and acute congestive heart failure (Cristóbal et al. 2022; Espadas-González et al. 2023). This discrepancy suggests that the inflammatory response in dogs with leptospirosis differs of those reported in humans with cancer or cardiovascular diseases, or even in dogs with other conditions as previously hypothesized. This discrepancy can be attributed to the different immune responses that play a role in these pathologies. In the case of leptospirosis (Novak et al. 2023; Prapong et al. 2022), a humoral immune response has been described, unlike in cancer or cardiovascular disease where the primary response is mediated by cellular immunity (Dada et al. 2023; Liberale et al. 2020; Lou et al. 2005).

In conclusion, an increase in the NLR is observed in groups of sick animals, suggesting its potential as an indicator of systemic inflammation in canine leptospirosis. The PLR decreases in groups of dogs with leptospirosis that die, which could be a negative prognostic factor, although more studies are needed. The SII only increases in groups of dogs with leptospirosis that survive, this could be indicative of morbidity prognosis but not mortality. This is the first study in veterinary medicine that studies the NLR, PLR and SII ratios in canine leptospirosis. These parameters are affordable and easily obtained when a hematology machine is available. Hence, we suggest the use of these ratios as a routine analysis in dogs affected with *Leptospira spp*. More studies would be needed to better define gold standard cut-off values and usefulness of these ratios.

## Electronic supplementary material

Below is the link to the electronic supplementary material.

Supplementary Material 1

## Data Availability

No datasets were generated or analysed during the current study.
