# Supplementary material for: Determination of neutrophil-to-lymphocyte ratio, platelet-to-lymphocyte ratio and systemic immune-inflammation index in dogs with leptospirosis
Source: Vet Res Commun. 2024 Sep 10;48(6):4105–11. doi: 10.1007/s11259-024-10469-y (PMC11538265; doi:10.1007/s11259-024-10469-y)
Supplement: Supplementary file 1 — Supplementary Material 1 [file 11259_2024_10469_MOESM1_ESM.pdf]

IDENTIF	NEUTR/LINF	PLAQ/LINF	IIS	MUERTE	SHPL
P 8637_2	35,08	286	5016440	NO	SÍ
17735	2,42363294	10,1822753	392,323067	NO	SÍ
P13822_1	4,61538462	226,153846	1356923,08	NO	NO
17794	11,7683616	81,3559322	1694,64407	NO	SÍ
13140	28,8928571	142,857143	3467142,86	NO	NO
8687	3,59556787	67,0360111	870127,424	NO	NO
P 8637_4	7,45454545	98,1818182	805090,909	NO	SÍ
5326	9,08108108	104,324324	1752648,65	NO	NO
P 10269_2	12,5398139	49,6453901	614450,879	NO	SÍ
P 10521_1	4,45579268	113,313008	993641,768	NO	NO
P12974_1	11,299435	246,892655	4937853,11	NO	NO
P 11924_1	14,5299145	182,051282	3094871,79	NO	SÍ
P11336_1	28,7783505	8,86597938	494987,629	NO	SÍ
18088	15,5	131,578947	1550	NO	SÍ
P 7173_14	3,53953488	175,348837	1334404,65	NO	SÍ
14382	10,4054054	134,459459	2070675,68	NO	NO
13244	8,84065934	108,791209	3500901,1	NO	NO
2414	34,8623853	72,9357798	5543119,27	NO	NO
13183	8,93172691	22,0883534	491,24498	NO	NO
P11387_1	17,4137931	270,689655	2733965,52	NO	SÍ
P12638_1	6,50887574	80,4733728	1770414,2	NO	NO
P8637_1	3,31404959	193,38843	775487,603	SÍ	SÍ
2765	36,7924528	181,132075	7064150,94	SÍ	NO
8253	8,99470899	56,6137566	962433,862	SÍ	SÍ
1521	16,9491525	5,93220339	118644,068	SÍ	NO
P13032_1	7,96460177	385,840708	3472566,37	SÍ	NO
17480	10,9933555	64,1196013	2121,71761	SÍ	SÍ
8487	7,19172932	28,9473684	553763,158	SÍ	SÍ
P 7554_4	14,4067797	25,4237288	432203,39	SÍ	SÍ
18092	10,029661	85,1694915	2015,96186	SÍ	SÍ
P 7895_1	8,33333333	10,6658924	459166,667	SÍ	NO
P 10479_1	6,69796557	3,4428795	147355,243	SÍ	SÍ
14162	12,4979079	67,7824268	2024661,09	SÍ	SÍ
P 11099_1	38,0909091	184,090909	3085363,64	SÍ	SÍ
18503	18,4041096	280,821918	7545,68493	SÍ	SÍ
P8711_3	14,5232558	131,395349	1641127,91	SÍ	SÍ
